# Prehospital Release of Patients After Treatment in an Anesthesiologist-Staffed Mobile Emergency Care Unit

**DOI:** 10.1001/jamanetworkopen.2022.22390

**Published:** 2022-07-20

**Authors:** Johannes Bladt Andersen, August Emil Licht, Tim Alex Lindskou, Erika Frischknecht Christensen, Louise Milling, Søren Mikkelsen

**Affiliations:** 1 Department of Clinical Research, University of Southern Denmark, Odense, Denmark; 2 The Prehospital Research Unit, Region of Southern Denmark, Odense University Hospital, Odense, Denmark; 3 Centre for Prehospital and Emergency Research, Aalborg University and Aalborg University Hospital, Aalborg, Denmark

## Abstract

**Importance:**

Prehospital treatment and release of patients may reduce unnecessary transports to the hospital and may improve patient satisfaction. However, the safety of patients should be paramount.

**Objective:**

To determine the extent of unplanned emergency department (ED) contacts, short-term mortality, and diagnostic patterns in patients treated and released by a prehospital anesthesiologist supervising a mobile emergency care unit (MECU).

**Design, Setting, and Participants:**

This retrospective cohort study used a manual review of prehospital and in-hospital medical records to investigate all living patients who were treated and released by an MECU in Odense, Denmark, between January 1, 2011, and December 31, 2020. Patients were followed up for 30 days after initial contact with the prehospital service.

**Main Outcomes and Measures:**

Primary outcome measures included unplanned contacts with the emergency department less than 48 hours after prehospital treatment and prehospital assigned diagnosis. Secondary outcomes consisted of mortality at 48 hours and 7 and 30 days.

**Results:**

A total of 3141 patients were identified; 384 were excluded and 2757 were included in the analysis. The median patient age was 40 (IQR, 14-66) years; 1296 (47.0%) were female and 1461 (53.0%) were male. Two hundred thirty-nine patients (8.7% [95% CI, 7.6%-9.8%]) had unplanned contact with the ED within 48 hours; this rate was doubled for patients with respiratory diseases (37 of 248 [14.9% (95% CI, 10.7%-20.0%)]). Fifty-nine of 60 patients who died within 48 hours of release had terminal illness. Excluding these patients, the mortality rates were 0.04% at 48 hours, 0.8% at 7 days, and 2.4% at 30 days. Two thousand sixty-one patients (74.8%) had primarily nondefinitive observational diagnoses.

**Conclusions and Relevance:**

The findings of this cohort study suggest that prehospital treatment and subsequent release at the scene is safe. One patient in 12 attended the ED within the ensuing 48 hours. However, for patients with respiratory diseases, this rate was doubled. Hospital admission could be avoided for some patients in the end stage of a terminal illness.

## Key Points

**Question:**

Is it pertinent and safe for a prehospital physician to treat and release patients in a mobile emergency care unit at the scene without hospital admission?

**Findings:**

In this cohort study of 2757 patients, 239 had unplanned contact with the emergency department within 48 hours of release after the prehospital contact. One patient without a terminal illness unexpectedly died within 48 hours after prehospital contact.

**Meaning:**

These findings suggest that treatment and release of patients is feasible and safe in many prehospital cases, with the added benefit that some patients with terminal illness may be released at the scene to die in familiar surroundings with their next of kin present.

## Teaser

This cohort study investigates the rate of unplanned contact with the emergency department within 2 days and the diagnostic pattern and short-term mortality of patients initially treated and released at a mobile emergency care unit.

## Introduction

Many patients treated in a prehospital setting do not wish to be transported to a hospital. Safe nonconveyance may be an attractive solution for such patients.^1^ However, even patients with low-acuity illness released at the scene by emergency medical technicians (EMTs) and paramedics may later be diagnosed with a time-critical condition.^2^

Since the 1980s, the Danish emergency medical service has developed from a system focused on rapid transportation of patients into a 3-tiered system offering advanced prehospital treatment. Ambulance personnel are not allowed to release a patient at the scene after treatment unless adhering to specific guidelines.^3,4^ Thus, the usual outcome after patient contact with an ambulance is the transport of the patient to a hospital.

The release of a patient at the scene is becoming more relevant to patients and health administrators.^1,5^ Owing to the increasing level of competency among prehospital services, in some instances even a patient with a high-acuity problem may receive prehospital treatment and subsequently be released, avoiding hospital transport.^6^

Paramedics or EMTs rarely release patients in the prehospital setting.^3,4^ However, a prehospital physician may make individual assessments concerning a patient and is less restricted when deciding to conclude treatment on-site instead of conveying the patient to a hospital. Thus, a safe nonconveyance strategy may be introduced in a larger variety of patients, even those with terminal illness.

In 2014, the outcome of patients treated and released by the mobile emergency care unit (MECU) in Odense, Denmark, was investigated.^6^ Among these patients, 0.2% died within 24 hours and 7% sought renewed contact with the emergency medical service or the emergency department (ED) within 24 hours. In the years that have passed since that study, both the diagnostic capabilities and the treatment capabilities of the MECU have expanded. Several new point-of-care technologies have been implemented that enhance the foundation for deciding to release a patient at the scene after treatment.^7,8,9,10,11^

We investigated whether patients initially treated and released at the scene had unplanned contact with an ED within 48 hours of the event. We also investigated the diagnostic pattern and short-term mortality of patients released at the scene after treatment by the MECU in Odense during a 10-year period.

## Methods

This population-based, retrospective cohort study assessed all MECU missions in Odense, Denmark, from January 1, 2011, to December 31, 2020. According to Danish legislation, register-based studies do not require approval by the scientific ethical committee. Approval for this study and the decision to waive informed consent was made by the Legal Department of the Region of Southern Denmark. The study followed the Strengthening the Reporting of Observational Studies in Epidemiology (STROBE) reporting guideline.

### System Setting

The prehospital system in Denmark is a tax-funded system without any immediate costs to the patient. It consists of 3 tiers: (1) an ambulance staffed by 2 paramedics; (2) 1 paramedic in a rapid response vehicle; and (3) a physician specialized in anesthesiology, either in a ground-based MECU or in a helicopter-based emergency medical service.^12,13^

The Danish emergency distress telephone number connects the caller with an emergency medical dispatcher. The dispatcher, usually a nurse or a paramedic, forwards the relevant prehospital resource according to the patient’s chief problem based on a criterion-based decision support tool.^14^

This study was performed in the catchment area of the MECU in Odense, Denmark. The MECU in Odense covers an area of approximately 965.3 square miles, servicing a population of approximately 300 000 people. Dispatch is initiated by either the emergency medical dispatch center or after a secondary request by ambulance personnel during contact with the patient.^14,15^ The MECU joins an ambulance in approximately 26% of all emergency dispatches with lights and sirens.^15^ The mean number of MECU missions per year is 3850. The MECU is staffed by 1 of 15 board-certified specialists in anesthesiology who are all part-time employees. The MECU anesthesiologist does not perform duties within the hospital while staffing the MECU. However, when not employed at the MECU, most of the working hours of the MECU anesthesiologists are spent within the hospital where they handle duties in the intensive care unit or the operating theaters or manage patients with the most severe illnesses in the ED. After each MECU run, patient characteristics, the prehospital diagnosis, and the mission outcome (patient admitted to hospital or released at the scene) are registered in the MECU database and each patient’s prehospital medical record.^13,15^

Patients at the hospital have their ED visits documented in the in-hospital medical records. A unique, 10-digit civilian register number assigned to each resident of Denmark allows for unambiguous identification and enables linkage with existing medical records.^16^ All living patients registered as released at the scene by the prehospital anesthesiologist and with a valid civilian register number were eligible for participation. Patients residing outside of the Region of Southern Denmark at the time of the incident were excluded.

### Data Sources, Patient Variables, and Outcomes

Data sources included prehospital medical records from the ambulances and the MECUs^2^ and in-hospital medical records. Patient variables included mission outcome, patient identification, date and time of the initial contact with the MECU (index date), prehospital diagnosis, and patient age and sex.

The primary outcome measure was unplanned contact with any ED, related to the initial contact with the Odense MECU within 48 hours of the initial contact. A further primary outcome measure was the prehospital diagnosis assigned according to the World Health Organization *International Statistical Classification of Diseases and Related Health Problems, Tenth Revision* (*ICD-10*), disease classification system.^17^ Secondary outcomes were mortality within 48 hours and 7 and 30 days of the initial MECU contact.

### Data Handling

Based on the MECU medical records, a data set was constructed with all missions with the outcome of living patient released at the scene after treatment.^15^ All prehospital and in-hospital medical records of the patients released at the scene after treatment by the MECU between January 1, 2011, and December 31, 2020, were manually scrutinized by 2 investigators (J.B.A. and A.E.L.). All follow-up was performed using the MECU medical records, the prehospital electronic medical records, and the in-hospital medical records.

Based on the MECU data set, prehospital and in-hospital medical records were searched for transports by ambulances and/or visits to an ED within 48 hours of the index date. The underlying cause of any subsequent contact with the prehospital service and/or the ED within 48 hours was established to ascertain whether the second contact was related to the previous contact with the MECU. If any such contact was related to the primary contact with the MECU, the initial incident was interpreted as a failed release at the scene. If a secondary contact was unrelated to the release of the patient to 48 hours before, the initial incident was interpreted as a successful release at the scene. Among patients who died within 48 hours after contact with the MECU, the medical records were reviewed for declarations of the patient having a terminal illness. In cases of doubt, a consensus was made among 3 investigators (J.B.A., A.E.L., and S.M.). Data were stored on an encrypted server, and all data handling was performed respecting the Danish and the European legislation concerning identifiable data.^18,19^

### Statistical Analysis

Data are presented as proportions presented with 95% CIs based on a binomial distribution or as medians and IQRs. Data were handled using Excel 2016 (Microsoft Corp), and Stata, version 17.0 (StataCorp LLC).

## Results

**Figure 1.**
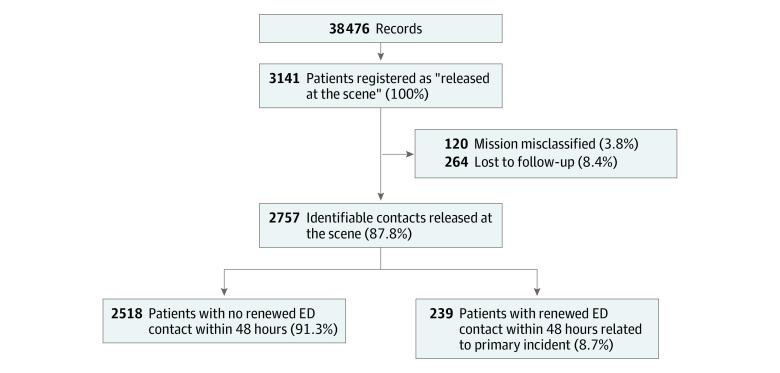
Study Flowchart ED indicates emergency department.

**Figure 2.**
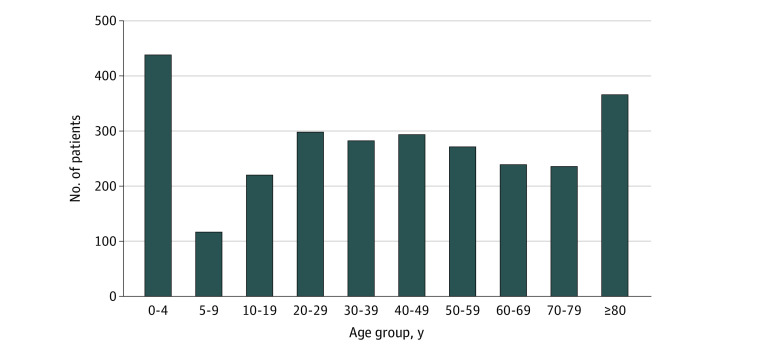
Patient Distribution According to Age

Of 3141 patients registered as released at the scene, 2757 were included in the analysis. The median age was 40 (IQR, 14-66) years; 1293 patients (47.0%) were female and 1461 (53.0%) were male. One hundred twenty patients were misclassified and 264 were lost to follow-up because they resided outside the Region of Southern Denmark. Details are provided in the study flowchart in Figure 1. The age distribution of the 2757 patients eligible for analysis is provided in Figure 2. Among the 2757 patients included in the analysis, 239 had hospital contact related to their prior MECU contact within 48 hours of the initial contact. In 24 cases of renewed contact with the ED within 48 hours, the patient’s contact with the ED was not related to their primary contact with the MECU. Of the 239 patients who had an unplanned contact with the ED related to the primary contact with the MECU, 227 attended the somatic ED, and 12 attended the psychiatric ED. The total rate of renewed contact of patients initially released at the scene after treatment by the MECU was 8.7% (95% CI, 7.6%-9.8%), with varying rates according to the initially assigned prehospital diagnoses. Most commonly, patients with respiratory diseases (*ICD-10* chapter X) had subsequent contact with the ED, occurring in 37 of 248 cases (14.9% [95% CI, 10.7%-20.0%]). Of note, 19 (51.3%) of these 37 patients were advised to accept transport to the hospital but refused.

The most common prehospital diagnoses were found within the *ICD-10* chapters XVIII (symptoms, signs, and abnormal clinical and laboratory findings, not elsewhere classified [n = 386]), XIX (injury, poisoning, and certain other consequences of external causes [n = 444]), and XXI (factors influencing health status and contact with health services [n = 1231]). These 3 diagnosis groups included 2061 (74.8%) of the 2757 patients in the study population. The distribution of the prehospital diagnoses assigned to the patients by the MECU can be seen in the Table.

**Table.** Distribution of Patients Within *ICD-10* Chapter Diagnoses and Rates of Unplanned Contact With the Emergency Department

*ICD-10* chapter^a^	Diagnosis group (code)	Patients released at the scene, No. (%)	Unplanned contact within 48 h, No. (%) [95% CI]
I	Certain infectious and parasitic diseases (A00-B99)	9 (0.3)	1 (11.1) [0.3-48.2]
IV	Endocrine, nutritional, and metabolic diseases (E00-E90)	187 (6.8)	11 (5.9) [3.0-10.3]
V	Mental and behavioral disorders (F00-F99)	95 (3.4)	6 (6.3) [2.4-13.2]
VI	Diseases of the nervous system (G00-G99)	67 (2.4)	7 (10.4) [4.3-20.3]
IX	Diseases of the circulatory system (I00-I99)	65 (2.4)	4 (6.1) [1.7-15.0]
X	Diseases of the respiratory system (J00-J99)	248 (9.0)	37 (14.9) [10.7-20.0]
XI	Diseases of the digestive system (K00-K93)	8 (0.3)	0 [0-36.8]
XIII	Diseases of the musculoskeletal system and connective tissue (M00-M99)	14 (0.5)	3 (21.4) [4.7-50.8]
XIV	Diseases of the genitourinary system (N00-N99)	3 (0.1)	0 [0-97.7]
XVIII	Symptoms, signs, and abnormal clinical and laboratory findings, not elsewhere classified (R00-R99)	386 (14.0)	29 (7.5) [5.1-10.6]
XIX	Injury, poisoning, and certain other consequences of external causes (S00-T98)	444 (16.1)	22 (5.0) [3.1-7.4]
XXI^b^	Factors influencing health status and contact with health services (Z00-Z99)	1231 (44.6)	119 (9.7) [8.1-11.5]
All	NA	2757 (100)	239 (8.7) [7.6-9.8]

Abbreviations: *ICD-10*, *International Statistical Classification of Diseases and Related Health Problems, Tenth Revision*; NA, not applicable.

^a^ Chapters that did not contain any patients are not shown.

^b^ Patients diagnosed within chapter XXI were primarily diagnosed with observation for unspecified disease (611 [49.6%]), observation following transport accident (419 [34.1%]), and other accidents (149 [12.2%]).

In the study population, 60 patients died within 48 hours after their prehospital release at the scene. Eighty-two patients died within 7 days, and in total 123 were dead within 30 days. The overall cumulative mortality rates were 2.2% (95% CI, 1.7%-2.8%) at 48 hours, 3.0% (95% CI, 2.4%-3.7%) at 7 days, and 4.5% (95% CI, 3.7%-5.3%) at 30 days.

Fifty-nine of the 60 patients who died within 48 hours after contact with the MECU had previously been declared to have a terminal illness by the patient’s primary care clinician or the hospital department due to chronic or malignant disease. These 59 patients were all released to their homes to receive end-of-life care surrounded by their next of kin. One patient was unexpectedly found dead the day after being treated and released by the MECU. When excluding the terminally ill patients, 1 patient died within 48 hours; 23, within 7 days; and 64, within 30 days. The following cumulative mortality rates were recorded: 0.04% (95% CI, 0-0.2%) at 48 hours; 0.8% (95% CI, 0.5%-1.2%) at 7 days; and 2.4% (95% CI, 1.8%-3.0%) at 30 days.

## Discussion

The main finding of this study is that 1 patient in 12 released at the scene after treatment had unplanned contact with the somatic or psychiatric ED within 48 hours of the initial contact with the MECU. Fifty-nine patients (2.1%) released at the scene were in the end stage of a terminal illness and were released to die in their own home surrounded by family as part of end-of-life care. Excluding these patients, the short-term mortality after prehospital treatment and subsequent release at the scene was low in this 10-year observational cohort study.

### Other Studies

Compared with a previous study conducted in our setting,^6^ the MECU in the present study had fewer missions and a released fewer patients. Since the previous study was performed, the Danish prehospital organization has changed. Emergency medical dispatch centers staffed by health care workers have taken over the task of prioritizing the prehospital resources. The present system tends to triage patients with a high risk of admission and death to higher tiers of the emergency medical service.^14^ This indicates that the MECU’s target population in this study has a higher severity of illness than previously. Prioritizing missions to patients with an increased degree of illness may explain why the MECU in Odense has conducted fewer missions than previously (10.5 vs 13.5 missions per day) and has reduced the number of patients released at the scene (10.6% to 8.1%).

In other studies,^20,21^ nontransport rates after prehospital treatment by physicians have been shown to vary from 8.0% to 15.7%. These studies, however, typically assess patients across an entire prehospital service rather than only those seen by a prehospital physician. The rates of nontransport of patients after contact with ambulances staffed by EMTs and paramedics vary from 3.7% to 93.7% in very selected cases.^22,23,24^ In another Danish study from a different health region,^22^ consultations between EMTs or paramedics and a physician at the emergency medical dispatch center resulted in the release of more patients with noncritical presentation at the scene with high patient satisfaction and without compromising patient safety.

Ebben et al^25^ conducted a comprehensive review of studies of nonconveyance in ambulance care. In their review, renewed contact with the ED after prehospital release at the scene occurred in 4.6% to 80.1% of patients. However, it is difficult to compare these results owing to the lack of uniform data on the patient population’s diagnostic pattern across these studies.

### Unplanned Contacts and Diagnostic Patterns

Patients assigned an *ICD-10* diagnosis within chapter X (diseases of the respiratory system) had a renewed contact rate of 14.9% compared with the overall rate of 8.7%, indicating that caution should be exercised when treating these patients. Of all the 248 patients with diseases of the respiratory system released at the scene, 37 had unplanned contact with the ED within 48 hours. Of these 37 patients, 19 (51.3%) had been advised to accept transport to the hospital by the MECU but had refused this offer. Respecting the patient’s autonomy may lead to cases in which a prehospital physician may consider releasing a patient after treatment to be inappropriate but must respect the wishes of the patient.^26,27^ In our setting, most patients released at the scene were released after the assessment of the anesthesiologists present. Apart from patients with chronic obstructive pulmonary disease, it was very rarely that patients did not follow the suggestions made by the anesthesiologist regarding transportation to the hospital.

Højfeldt et al^6^ concluded that caution should be exercised when releasing patients at the scene after motor vehicle crashes. They found that 13% of these patients were seen again in the ED. During our observation period of 10 years, 419 patients were treated after motor vehicle crashes (Table). We observed similar numbers for renewed ED contact, and the previously issued caution regarding the release of patients treated after crashes is therefore still valid.

Of all patients released at the scene, 1231 (44.6%) were assigned an *ICD-10* diagnosis within chapter XXI (factors influencing health status and contact with health services). This is a marked increase compared with the previous report of 28%.^6^ This increase could be a cause for concern; although these patients have overall low mortality rates, their sheer numbers, in essence, contribute to a high number of deaths.^28^

### Mortality

Among all 2757 patients, 60 (2.2%) died within the 48-hour follow-up period. Of these, 59 were declared to have a terminal illness by their primary care clinician or a hospital department. These patients were purposely released at their home or nursing home for terminal care in well-known surroundings. Prehospital end-of-life care is associated with increased quality of life in patients with terminal cancer,^29,30^ who often prefer to die in their own homes.^24^ Releasing patients at the scene enables the prehospital services to respect and accommodate patient and family wishes and improve patient care. In our study, 1 unexplained death (0.04%) occurred within 48 hours. The mortality of the total prehospital population is only sparsely investigated internationally. Even more sparse are reports of mortality after prehospital treatment and release. However, in patients with an opioid overdose, mortality was reported in 0.08% of cases after treatment performed by ordinary ambulance personnel.^31^

Other studies focusing on the total patient population hospitalized after treatment in an ordinary ambulance^32^ found a cumulative 30-day mortality rate of 4.7% (95% CI, 4.6%-4.8%). The 30-day mortality rate among all patients attended by MECU was 5.7% (95% CI, 5.4%-6.0%).^15^

After excluding the patients with terminal illness, the 30-day mortality rate for patients released at the scene was 2.4%. Our study thus suggests that the release of patients after prehospital treatment is a relatively safe practice.

### Strengths and Limitations

Strengths of this study include the equal access to prehospital services in Denmark, which ensures that the socioeconomic status of patients did not affect patient inclusion in our study. This combined with the population-based study design, precise eligibility criteria, and thorough follow-up reduced the risk of selection bias.

This investigation was performed within a prehospital system based on EMTs and paramedics supported by an on-scene physician. Our results are thus not valid in emergency medical systems based solely on EMTs and paramedics. Furthermore, the MECU is staffed by specialists in anesthesiology rather than emergency physicians. This is in part due to a historic Danish tradition, because in Denmark, the specialty of emergency medicine evolved long after the concept of prehospital physicians did. Thus, the MECUs are primarily intended to treat patients with the most severe illness by offering tracheal intubation, ventilatory support, and circulatory support in a prehospital setting on par with the initial treatment that can be offered in the intensive care units within hospitals.

One major limitation in this retrospective cohort study is that no information was available concerning the patients’ perspectives of being released at the scene. Other studies investigating patients with chronic obstructive pulmonary disease, however, point toward patients considering it appealing to avoid admission to the hospital.^1^

Patients were lost to follow-up if living outside the Region of Southern Denmark, because it was not possible to gain access to their medical records. Although their general distribution of diagnoses did not differ substantially from the patients residing in the Region of Southern Denmark, this loss of follow-up may bias our results toward fewer patients registered as seeking unplanned ED contact.

## Conclusions

In this cohort study, we found a low mortality rate and a low frequency of unplanned ED contacts among patients released in a prehospital setting. A safe nonconveyance strategy with individual assessments by prehospital physicians may benefit patients and allow for end-of-life care in patients’ homes; however, this approach requires a paradigm shift. This could be enhanced by stressing that a call to an emergency medical dispatch center is not in itself an indication for transport to a hospital, but rather an indication that a patient needs thorough examination and diagnostics at the scene.
